# "The Hospital" Nursing Mirror

**Published:** 1897-03-06

**Authors:** 


### "The Hospital" Nursing Mirror.

Being the Nursing Section of "The Hospital."

[Contributions for this Section of "The Hospital" should be addressed to the Editor, The Hospital, 28 & 29, Southampton Street, Strand, London, W.C., and should have the word "Nursing" plainly written in left-hand top corner of the envelope.]

### News from the Nursing World.

### THE QUEEN'S COMMEMORATION.

The Prince of Wales has become a patron of the Queen's Commemoration Fund in aid of the Queen's Jubilee Institute for Nurses. The fund has now reached £28,534.

### GUY'S HOSPITAL INSTITUTE FOR TRAINED NURSES.

There is no more successful institution in London than the Guy's Hospital Institution for Trained Nurses, and it is a pleasure to read the annual report just received. During the past year the organisation has been remodelled, and a body of managers constituted consisting of four *ex-officio* members, the president and treasurer, the superintendent, and the matron of the hospital, with three representatives of the governors and two representatives of the medical and surgical staff elected annually. The fees earned by nurses last year amounted to £6,798, "by far the largest total yet recorded." At the end of 1895 the institution had standing to its credit with the Royal National Pension Fund for Nurses a sum of £2,626, and to this an addition of £622 has been made during the past year in the shape of quarterly payments to secure for each nurse a pension of £11 5s. at fifty years of age. The profits of the year's working have allowed a bonus division at the rate of £4 a share to the twenty-three nurses entitled to participate, this money being invested with the Pension Fund to secure a pension for the nurse at fifty-five, when, by the rules, she retires from active service. Arrangements have been made for the reception of nurses into one of the M.A.B. hospitals for six months' special training in the nursing of infectious diseases. Two maternity nurses are maintained at the cost of the institution, and these have done excellent work during 1896 in the poor districts of Southwark and Bermondsey. Subscriptions to the Maternity Samaritan Fund, which provides necessary food and medical comforts for destitute mothers, are asked for and much needed. Contributions of money or clothing should be sent to Miss Sarah A. Swift, lady superintendent of the institution, 14, St. Thomas's Street, S.E.

### DISTRICT NURSES FOR READING.

After the comparative merits of a local old-age pension scheme, a convalescent home, a college of music, and various other suggestions for the suitable commemoration of the sixtieth year of the Queen's reign had been discussed at a public meeting at Reading, the claims of the sick poor were convincingly put forward by Canon Garry. Ultimately an amendment was passed: "That in the opinion of this meeting the formation of a local institution for nursing the sick poor in their own houses would be a suitable manner of commemorating the sixty years' reign of Her Majesty, and that donations and annual subscriptions in aid of that object be immediately solicited." A large and representative committee was then appointed.

### THE NEW NURSES' HOME, WORCESTER INFIRMARY.

The Countess of Coventry in February laid the foundation-stone of the new home which is now in course of erection for the Worcester Infirmary nurses. The building will be three stories in height, and is to contain 32 bed-rooms for sisters and probationers. The site is close to the infirmary itself, with which the home is to be connected by a covered way. The progress of the work will be watched with much interest by the infirmary nurses, who are already looking forward with very pleasant anticipation to the day when their new quarters will be ready for occupation.

### NEWS FROM SOUTH AFRICA.

We are glad to hear that Miss Vacher, who, it will be remembered, succeeded Sister Henrietta as Matron of the Kimberley Hospital, and was obliged to give up her appointment on account of her health, is now much better. She is not, however, well enough to return to nursing work for the present. Miss Cowan, who is now matron at Kimberley, was charge nurse there in Sister Henrietta's time. Five of the seven nurses who went to Kimberley to join Miss Vacher still hold posts there as ward sisters. Of the other two, one is matron of a small hospital in the Transvaal, and the other has been invalided home.

### NURSES "A LA MODE."

Two excellent contributions, one from "One of the Public who Likes Fair Play," the other from "A 'Trained' but not 'New' Nurse," will be found in the March number of *Nursing Notes* on this much debated matter, both of which may be commended to Lady Priestley's attention. The nurse's letter touches especially upon a point which certainly the public are apt to overlook when blaming the modern private nurse for not fulfilling their ideal of what she should be. And this is the backwardness which the public almost universally manifest to report an unsatisfactory nurse to the institution from which she comes. Doubtless it often arises from natural reluctance to bring censure upon a woman whose daily bread depends upon her giving satisfaction to her employers, but it is a very cruel kindness, this, to the nurse herself no less than to the public. The writer states an obvious fact when she says "the general public lacks backbone, it prefers to sit still and throw mud at the whole community of private nurses; it finds universal abuse of a class so much safer and easier than the formal censuring of an individual. Hence the incompetent or untrustworthy nurse is left at large because the general public permits her to pass unrebuked. . . . When employers of nurses report truthfully on the quality of the service which they have received in return for their payments in hard cash, then, and then only, will they be protected from the type of woman who brings discredit on many a noble nurse."

### UNSUITABLE APPLICATIONS.

Some little time ago a correspondent wrote to us asking for certain particulars about a hospital, mentioning that she had written to the matron respecting a vacancy for a probationer, but had received no reply. We have taken the trouble to inquire into the reason of the silence on the part of the matron, who is a lady who has invariably a good reason for what she does or leaves undone, and find that the application was made on a postcard, and worded in a somewhat offhand manner, leaving an impression that the applicant would scarcely be a likely candidate. To people who are accustomed to observe character minutely, a little matter of this kind will reveal a good deal, and a careless and slap-dash way naturally gives the impression of an undesirable lack of soberness and thoughtfulness. The would-be nurse will find her desire for information invariably met with attention and courtesy if she herself writes a courteous letter. And in writing to a voluntary hospital she may well also remember to enclose a stamped envelope for reply.

### NATIONAL UNION OF WOMEN WORKERS.

An interesting series of lectures is being given under the auspices of the National Union of Women Workers, Berners Street, at the Portman Rooms on Fridays at half-past eleven. The "History of the English Poor Law" has been dealt with by Miss Sophia Lonsdale, a guardian of the Lichfield Union, during February, and on Fridays in March, Miss K. Bannatyne is lecturing on "The Care of Women and Children under the Poor Law."

### TRAINED NURSES' CLUB.

Frequenters of the Trained Nurses' Club, Buckingham Street, will be glad to hear that Mrs. Nichol, the ever-kindly and courteous secretary, who has been seriously ill is now better, though forbidden to leave her room till the weather is warmer. The Committee of the Club have voted her a holiday. Whenever Mrs. Nichol returns to her post she is certain to meet with a very warm welcome from many who have missed her during the past weeks.

### JOTTINGS FROM THE PUNJAB.

Of the four military stations in the Punjab to which nursing sisters are sent Umballa is one of great importance, for a large body of troops is always quartered there. The ordinary garrison consists of one British cavalry and three infantry regiments, but this year there are the 16th Lancers, the Black Watch (Royal Highlanders), as well as the Surrey and Bedfordshire regiments, so that there are sure to be a good many sick "Tommies" claiming the care and attention of the sisters. Umballa is a healthy station, and epidemics of cholera or enteric are rare. The Station Hospital where the nursing sisters work is almost in the centre of the cantonment. Their quarters are large and comfortable. Two sisters are attached to the hospital, and a deputy lady superintendent. One of the sisters, Miss Hallowes, is just going to be married to the Rev. F. Ferrier, senior chaplain of the Kirk of Scotland. The wedding is to take place in St. Paul's Church, and the bride will be given away by General Symonds, C.B., commanding Umballa. Sister Hallowes received her training as a nurse at the Birmingham General Hospital, and joined the Indian Nursing Service in 1892.

### LEEDS DISTRICT NURSING ASSOCIATION.

A truly wonderful impetus is being given to district nursing in every part of the country, and the number of localities where "the Queen's Commemoration" has resolved itself into schemes on behalf of the district nursing associations is little short of astonishing. In Leeds the District Nursing Association, affiliated with the Q.V.J.I., seems likely to benefit greatly this year. At present the association supplies nurses to twelve districts, employing altogether fifteen nurses, but adequately to spread the advantages of skilled nursing over the whole town it is estimated that ten more districts must be formed, and to carry out this many influential people in Leeds are lending the weight of their support and approval.

### TWO LECTURES FOR NURSES.

Dr. Henry Kenwood gave an interesting lecture on "Home Sanitation," on Friday, February 26th, at eight p.m., at the offices of the Royal British Nurses' Association, 17, Old Cavendish Street, W. The lecture, which was accompanied by lantern illustrations, was based on the essentials requisite for a healthy dwelling-house, a subject comprising ventilation, drainage, and water supply.—At the Victoria Commemoration Club for Nurses on Tuesday last, Mrs. Alting-Mees lectured on "The Chemistry of Food," before a large and appreciative audience. The extensive and complex subject was handled in a very lucid and able manner by the lecturer.

### NOTICE TO CORRESPONDENTS.

Want of space compels us, week by week, to leave many of our correspondents' queries unanswered. We trust they will understand that each will receive attention in due course, and will be replied to in "Notes and Queries" column as soon as possible. It is a great satisfaction to feel that our readers find the information we are able to give to them in this way of value, and take this opportunity of thanking one and all for the many kind expressions of appreciation which frequently reach us in these letters.

### SHORT ITEMS.

A successful concert was given in aid of the Sunderland District Nursing Fund the other day. During an interval Dr. Morgan pleaded the cause of the fund, and asked for an endowment of £10,000 to enable the poor of Sunderland to be well and adequately nursed. —It seems probable that a County Nursing Institution will be started at Truro in commemoration of the Queen's reign.—A "discussion, meeting, and soirée" was held at Hackney Town Hall on March 1st in aid of the North-Eastern Hospital for Children, by the "Hackney Society in aid of the Children's Hospital."—Miss Lillie Waddington, Matron of the Bootle Borough Hospital, contributed an article in reply to Lady Priestley to the *Woman's Signal* for February 18th. —Miss Hetherington has resigned the post of Matron to the City of London Hospital for Diseases of the Chest, Victoria Park, after a service of twelve years.—Miss M. M. Traill Christie, M.B., B.S., has been unanimously re-elected to the post of second resident medical officer to the Greenwich Union Infirmary and Workhouse.

### Lectures to Surgical Nurses.

By H. A. Latimer, M.D. (Dunelm), M.R.C.S. of Eng., L.S.A. of London, Consulting Surgeon, Swansea Hospital; President of the Swansea Medical Society; Lecturer and Examiner of the St. John Ambulance Association, &c.

### II.—Meanings of the Terms "Symptoms" and "Signs"—Wounds Defined: Their Varieties and Methods of Repair.

The medical man in investigating a case groups the various systems of the body and regards them separately under the various businesses which each part of it has to perform. Thus he regards the nervous system, the circulatory, breathing, digestive, and excretory systems one by one; he reviews them minutely, and examines them by methods which he has acquired as a student; and if he does this thoroughly it is very unlikely that things which are going wrong in any parts under examination will be overlooked by him. You would do well to regard the body from the same point of view, and to think over symptoms which present themselves to you, whilst noting them for the doctor's benefit.

Now it will be well if I advise you what is meant by a symptom, and what varieties there are of symptoms. By a symptom is meant the expression by word of mouth, or by some change in the appearance of the part, or by the appearance of some new sign of formation which is indicative of disease. Symptoms, looked at from this point of view, are disturbances of natural functions in the body. It is the natural function of the nerves to carry sensation, but when they give us that sensation which is known as pain, we have a symptom. It is natural for the breathing organ to resent the introduction of dust, or of an irritating smoke, by cough; but cough becomes a symptom when it is persistent, without apparent reasons of this kind. Symptoms are of two kinds: subjective, or such as can only be felt by the persons suffering from them; and objective, when they are apparent to other people. To make my meaning plain, I would say that we have a subjective symptom when the patient describes to us pain of a certain kind flowing from the spinal column; the symptom becomes objective when the spinal column has yielded to disease, and we see it curved in one or another direction. Some symptoms are further known by the name of signs, but such symptoms are few. A sign is a symptom which points definitely to one disease, and proclaims that disease, and no other, as the one from which the patient is suffering. Thus many of the rashes which appear in the special fevers are said to be signs, having peculiarities of their own which mark them as indicating a special disorder. One may have been attending for some eight or nine days a case of fever, obscure in its nature, but without sufficient indications to warrant one in saying that probably it is typhoid fever. About this time some small spots may make their appearance—scattered, rose red, slightly raised, fading on pressure but reappearing afterwards, lasting two or three days only. These spots will clinch the diagnosis; we can now confidently proclaim the case to be one of typhoid fever. On the appearance of such spots one would say triumphantly, "here is a sign," it points definitely to one thing, and means that only.

Passing from these general considerations, we will start directly on our work, beginning with the consideration of wounds. A wound may be defined as a tear, breakage, or division of any part of the body. There are two great divisions of the subject into "open" and "subcutaneous" varieties, by which, of course, is meant in the first place a tear of the skin and parts underneath it; in the second, the tear of parts beneath the skin, whilst the structure remains intact. Wounds are accompanied, like most other disturbances, by local and constitutional symptoms; that is, they disturb the part involved in the breakage itself, and the body generally is disturbed by the pain or fever or illness which follows the injury received. The severity of these symptoms is due to injuries of important parts involved, such as blood-vessels, nerves, bones, or internal organs. The healing of open wounds is much complicated by the introduction of germs, or bacteria—organisms which are always floating about in the atmosphere, and which not only settle on wounds of their own accord, but are brought there by dirty hands, dirty instruments, and faulty dressings. These germs, you will find, are rightly considered nowadays to be at the bottom of much of the inflammation which accompanies wounds, and I should like to fix in your mind what is meant by the word germ. A germ is well defined in the dictionary as "the rudimentary form of an organism; the seed bud; that from which anything springs." As I shall have again to deal with this matter of germs rather fully, later on, I will postpone the consideration of them for the present. Returning now to wounds, I would have you notice that the open wounds are known by different names, according to their peculiarities: we have the incised wound, when the skin has been cut by a sharp instrument: the lacerated one, when the parts have been torn unevenly; the contused one, when there is bruising; and the wound is said to be punctured when its depth exceeds its length, as in the case of a stab. These various wounds have different methods of healing, and are more or less dangerous according to the constitutional state of the person upon whom they are inflicted, and the treatment he receives afterwards; but they have one especial liability to danger, and that is that, being open and exposed to the air, they afford a favourable site for the settlement and injurious influence of germs. Subcutaneous wounds are free from this latter danger, as no germs can gain admission to them, they generally heal kindly, and are for the most part without the graver consequences which attach to the open wounds. Contusions or bruises are oftentimes tedious in their healing process because there has been a great deal of crushing, pulping, and tearing of parts, with escape of blood from the smaller blood-vessels. Punctured wounds are especially dangerous because of the liability to injury of deep seated and important organs which are out of sight. You will have many opportunities of observing the process of healing of wounds—the so-called methods of repair. There are three great modes by which they heal, known as the first, second, and third "intentions." Taking an incised or clean cut wound as our example: if the edges of the cut part adhere to each other there and then, and the wound heals right off, it is said to have united by the first intention. If the same wound remaining open becomes healed by the springing up of small red points of flesh called "granulations," which, when they have arrived at the level of the surrounding skin, become covered with skin by the extension of the same over these granulations, we are said to have union by second intention; and when these granulations in an open wound, springing from the sides and bottom of it, adhere to each other, and so close the wound, we have what is known as union by the third intention. There is a fourth mode of healing which I am thankful to say we seldom see nowadays; that is when one or more of these processes go on concealed from our view by a scab or crust, that is said to be union under a scab. I hope you will not have any opportunities of seeing this kind of healing process at all.

### Post-Graduate Clinics for Nurses.

By a Trained Nurse.

### IV.—THE EVOLUTION OF THE PRIVATE NURSE.

I concluded my last paper by citing one or two instances where friction and trouble resulted in private houses from nothing more serious on the part of the nurses engaged there than a want of *savoir faire* and knowledge of the world. The question may be asked as to how a nurse may acquire this inestimable knowledge, and how she may overcome the many difficulties which beset her path when she emerges from the Training School, where she has been safeguarded and looked after, and where she has not been to any great extent thrown on her own responsibility.

Now, so far as a knowledge of the world is concerned, the home life and early training of a young woman count for much. In the Training School every sort and condition of femininity is met with, a very common type being that of the girl who has lived her first twenty-four years in a quiet country village, having had, perhaps, a home education.

A girl with a history like this, at the end of her training is by no means well equipped for private nursing. True, she has had three years in the world of Hospital; but this is a world more or less of one kind—a homogeneous world—and she will meet with difficulties in private nursing that are of many different kinds. Again, we find the probationer educated at high school and college, who, by the time she takes her certificate, has a knowledge of people and things, has learned to find her own level and to estimate that of others in a way which will be most valuable to her in a private nursing career. It has always appeared to me that one important point to remedy so far as nurse training goes is the age at which the probationer enters the Hospital. The age-standard is too high at present; and certainly a very strong argument in favour of a lowered age at entrance, and one which would help in the evolution of successful private nurses, lies in the fact that it is far easier to train in definite and methodical habits the girl of twenty-one than the woman of twenty-four. And it can hardly be denied that the years spent by a girl in "waiting" to enter hospital are spent often in a desultory and aimless fashion, which militates against her acquiring that perfect trained faculty which the standard of to-day demands in nursing, as in other professions. Another great help in the evolution of the private nurse, which at the same time appears practical and feasible, would be the adoption of some plan whereby in the nursing institutions connected with our Training Schools it might be arranged that the nurse during her first year or six months of private nursing should be sent by preference to those cases where two nurses are employed. In this way she would be introduced more gently and by degrees to the ways and methods of private nursing—for the second nurse in the case should be one who is experienced in this branch of nursing work. By so simple a plan the nervousness and trepidation natural to a nurse leaving the wards where she has been afforded so much help and support to go on her own responsibility among a set of strangers would be saved, and not only would the nurses of a Training School adopting such a plan assuredly gain a high reputation for skill and efficiency, but there would be fewer of the 'prentice hand mistakes of which we hear so much, but which are by no means to be wondered at since they are a perfectly natural and logical outcome of the prevailing error that anyone who understands nursing can undertake a private case.

An instance of how a lack of worldly wisdom may militate against the success of a nurse occurs to me here as coming under my observation in the house of a sick friend. One of her trained nurses made a careful stipulation when first she came that she was to have a certain time "off-duty" each day; my friends were perfectly willing to grant her every right and privilege, but their reverence for this nurse's wisdom and knowledge received a rude shock when they discovered that these important daily "outings" which allowed of no postponement or delay were taken for the purpose of visiting a mesmeriser who was "curing" the nurse of rheumatism by hypnotic suggestion! They felt as if they could hardly recommend such a nurse to their friends. In a case of this kind it would have been most valuable for the nurse in question to serve for a year under a senior private nurse, who might by her assistance and advice have preserved her from such a pitfall.

Again, what useful hints an experienced private nurse might give to another who emerges from hospital with fixed and unalterable views on ventilation; a nurse who translates the theory of fresh air and its necessity to the sick as given in the text-books with as much rigidity as does a Puritan a verse in the Bible arguing against amusement. This kind of nurse, without some saving grace and good advice, will assuredly get into trouble in private practice. For the average patient likes his fresh air in homœopathic doses, and he must have the theory of "inlet and outlet" instilled very gently into his daily consciousness. The nurse new to private work is too apt to quote her late ward, and relies on the precepts taught and practised there, with the blind reliance a devout follower of Mohammed places in his Prophet. But the nurse, in this question of ventilation, as in many other matters pertaining to the private sick-room, must temper the wisdom learnt of her Training School with a large admixture of worldly knowledge. And she will soon find that the incurable draughtiness of the average hospital ward will be most unpopular if introduced into the private sick-room. If, too, she have a real, not a superficial, knowledge of the laws of hygiene, she will recognise that less fresh air is needed for one patient relatively than is required when twenty to thirty persons suffering from different diseases—and this point makes all the difference—are collected together in one apartment. To learn to ventilate—not too well, but wisely—is one of the first accomplishments to add to the outfit of the private nurse.

### Presentations.

On leaving the Chelsea Infirmary for the Essex and Colchester Hospital, of which she was recently appointed matron, Miss Elizabeth Davies was presented by the staff with a valuable Dresden china tea service and a handsome silver clasp as a token of the esteem and affection in which she was held by everyone connected with the institution.

On Tuesday, February 23rd, a purse of money was presented at the Essex and Colchester Hospital to the matron, Miss Brough, on the occasion of her retirement after seven years' tenure of the office, during which time she reorganised the nursing department and introduced many other improvements. The presentation was made by the chaplain, the Rev. W. W. Labarte, and Dr. Wallace, senior physician, in the presence of the nursing staff, who had all, together with the chaplain and the medical staff and a few other friends, taken the opportunity of testifying their regard for the matron and their regret at losing her valuable services.

### A New Military Nursing Order.

The *Morning Post* states: The Secretary of State for War has approved of a new military nursing order, consisting of a reserve of nurses to supplement the regular nursing service of the army in time of war. In the regulations for the control of the order, which will be circulated in a few days, it is stated that the body will be known as the "Army Nursing Reserve." In time of peace it will be placed under the direction of a specially constituted committee, which has very appropriately been placed under the presidency of Princess Christian. In time of war the nurses, or as many of them as may be required, will be entirely under the control of the War Department. The newly-authorised body will consist of 100 or more nursing sisters, a certain number of whom may be detailed by the military authorities as acting superintendents.

A candidate for the appointment of nursing sister must be not under twenty-five or over thirty-five years of age, and must have had at least three years' preliminary service combined in a civil general hospital. She will be required to produce:—

(*a*) An extract from the register of her birth, or a declaration made before a magistrate by one of her parents or guardians giving the date of her birth.

(*b*) A recommendation from a person of social position (not a member of her own family) to the effect that the candidate's family is one of respectability and good standing in society, and that she is in every way a desirable person to enter a service composed of ladies.

(*c*) A statement signed by the candidate, showing whether she is single, married, or a widow, and whether she is a member of a sisterhood or society. The statement should also give particulars of the place and duration of her hospital training, which must, for at least twelve months of the time, have been in a civil general hospital where adult male patients receive medical and surgical treatment, and in which a staff of nursing sisters, under a matron, is maintained.

(*d*) Certificates of efficiency in medical and surgical nursing from the medical officers under whom she has served.

(*e*) A recommendation from the matron of the civil hospital at which she was trained, who must certify that she possesses the tact, temper, and ability qualifying her for appointment to the Nursing Service Reserve of the Army. Ability to speak one or more foreign languages will be considered an additional qualification, and will give preference in selection.

(*f*) A certificate from a qualified medical practitioner that she is in good health.

Candidates must state, in addition—

(*a*) What experience they have had in hospital supervision.

(*b*) What they personally understand by the duties of a superintendent, and the execution of medical orders.

When not required for service members of the Reserve will not be bound by any rules as regards dress or uniform, except that they must wear on the right breast the badge of the Army Nursing Service Reserve. When called up for Army nursing service members of the Reserve will become amenable to the ordinary regulations of the Army Nursing Service. On attaining the age of fifty, nursing sisters will cease to belong to the Reserve.

When called up for duty a nursing sister will receive pay at the rate of £40 per annum, and when performing the duties of acting superintendent she will receive extra pay at the rate of £20 per annum. On cessation of employment, nursing sisters—including acting superintendents—will receive a gratuity of £20. They will also receive a further gratuity of £10 for each year of service beyond the first, if at home, and of £20 if abroad. Fractions of a year will be calculated at the same rate. These gratuities are sanctioned subject to the following conditions:—

(*a*) The service rendered by a nursing sister must have been in all respects of a satisfactory nature and certified to by a responsible officer.

(*b*) The cessation of employment must have been due to causes beyond the nursing sister's own control.

(*c*) Any nursing sister relinquishing her engagement for reasons within her own control will forfeit her title to a gratuity—even though her services may exceed a year.

A special allowance in lieu of board and washing at the rate of 13s. a week at a home station, or of 3s. a day at a station abroad when rations in kind are not supplied, and of 3s. 6d. a week when they are supplied, will be granted to sisters of the Reserve when called up for duty. The other allowances at stations abroad, including the allowance for servants, will be at such rates, not exceeding those of a departmental officer of subaltern rank, as the Secretary of State for War may determine.

Nursing sisters when on service will receive a special allowance for the provision of clothing at the following rates: Annual clothing allowance abroad, £4 7s.; annual clothing allowance at home, £4; triennial winter cloak allowance, £2; triennial summer cloak allowance, £1 5s.

### East London Nursing Society.

The Lord Mayor presided over the annual meeting of the East London Nursing Society, held in the Egyptian Hall of the Mansion House, on Monday, March 1st. A large audience gathered to hear the report of the past year's work, and many of the nurses were also present.

The adoption of the report was moved by the Bishop of Stepney, who could speak with much personal knowledge of the work of the nurses, seeing that some thirty-two parishes in his diocese enjoy the benefit of their labours. The Bishop dwelt especially on two points connected with the society's special object—the work of "nursing the poor in their own homes." First the immense value of the co-operative work effected by the doctor, the nurse, the "assistant lady," who helps in the provision of necessaries for the patients, and other charitable societies, all the help required being thus instantly focussed on a particular case at the particular moment when it is needed. Secondly, he spoke strongly on the educative value of nursing, of the sensible knowledge of sanitary matters brought by the nurse into the homes of the poorest, of the simple domestic cookery she taught them, and the difference between a clean floor and a dirty one made apparent to them by her example. "If we wish to make a difference in the houses of the poor," concluded the Bishop, "we can do it in no better way than by increasing the numbers of district nurses."

Other speakers were the Rev. Prebendary Harry Jones, Sir Walter Phillimore (who made an eloquent little speech, pointing out how many were the cases which could be better dealt with in their own homes than in hospital, and touched on another very important matter, the desirability for increasing the present "humble salaries" of district nurses), the Rev. A. E. Dalton (rector of Stepney), and Sir W. H. Quayle Jones.

The East London Nursing Society was established so long ago as 1868, and was affiliated with the Queen's Jubilee Institute in 1891. Its work, as its name implies, lies wholly in the crowded East End, and there are now four centres, each with its superintendent matron, the nurses being resident each in her own district. Briefly, the value of the work done may be summed up in this extract from the report. During

1896 "we have nursed 4,984 cases, and can certainly count 3,466 recoveries, and probably many more among the 613 moved to hospital or elsewhere; amongst the 4,984 cases nearly 734 were men, who have thus been helped to regain strength, and with it the power of maintaining themselves and their families." The chief office of the society is at 49, Philpot Street, E.

### Nurses for the Plague.

We have received many inquiries in regard to nursing plague patients in Bombay, and it is clear enough that there are plenty of brave Englishwomen who, partly from their sympathy for suffering humanity of whatever nationality, partly from their *esprit de corps* and love of their work, are willing to risk their lives in carrying to that plague-stricken city all the help and comfort which skilled nursing can supply. The question however, as put to us by individual nurses, is not ought I to accept a post offered to me? but shall I pack up my traps and go? and it is clear that to these two questions very different answers must be given. If the Indian Government or the responsible hospital authorities in Bombay make a call for nurses we have no doubt whatever that the call will be readily, nay eagerly, responded to by the nurses of England. But at present there is no such call. We have made inquiries on this subject, and we are informed from official sources that there is at present no intention of sending out nurses from England, and that there seems no likelihood of such a course being adopted. We are also told that nurses are not at present wanted, and that there are a good many of them in India who would probably be available if they were required. This, be it observed, is the official view, and some may be tempted to say that it is only the official view. It must, however, be borne in mind that in India everything is official, and the official view is the only one that is worth the consideration of an outside individual. When the call comes let nurses go by all means. But the call has not yet come; and for nurses to invade Bombay on the chance of obtaining employment while those in India, familiar with its language and customs, have not yet been moved up to the scene of action would, in our view, be very foolish, although we quite admit the strong temptation to do so which is offered by the harrowing accounts which are continually arriving from those resident on the spot. We need hardly say that in almost every point India is different from England, or that the difficulty of language stands as a great barrier between the natives and all newly-imported nurses. No new comer in India would be of much use except within the hospitals, and the natives do not take kindly to these institutions. Taking the returns for February 8th, which we happen to have before us, it appears that while only 19 deaths from plague were reported from all the hospitals, 95 took place outside. At present, then, it would seem as if no serious attempt had been made to enforce the hospital isolation of cases of plague, and as for nursing the patients in their homes, that would be quite out of the question for such nurses as might go out from England.

So far as Bombay is concerned, the probability is that the worst is over. Plague seldom remains very long in one place. The lesson learned in Bombay, however, may perhaps lead to some alteration in the methods adopted in dealing with the disease in other places, and if any serious attempt should be made to fight plague by hospital isolation, and if the authorities should decide to introduce into these hospitals what we in England would understand by the term "a complete system of nursing," then there will be a great opening for nurses. But as we have already said, that time is not yet, and it must depend upon official action whether and when that time shall arrive.

### Nursing at the London Hospital.

The opening of the new Nurses' Home at the London Hospital, and the consequent increase in the staff, has at last made feasible a rearrangement of hours which Miss Lückes has for long been anxious to carry out. Briefly, the new arrangements provide that sisters are given a half-day once a week instead of once a fortnight, and every other Sunday, to 11 p.m., without going into the wards at all. The half-day of the preceding week may be taken on Saturday, and that night spent away from the hospital if desired. Staff nurses will in the future have a half-day once a fortnight, instead of once a month, from 1.30 p.m. to 10 p.m., and the whole of every fourth Sunday to 10 p.m., without going into the wards at all. In their case, too, the half-day may be taken on the preceding Saturday and the night spent away from the hospital. Probationers will be entitled to the whole day off duty once a month, not entering the wards on that day at all. Probationers on night duty as well as staff nurses will be granted a fortnightly day off. No nurse or probationer will now be required to enter the wards on the morning of the day they leave for their holiday. Nurses and probationers will be allowed to have breakfast in bed on their days off, a little luxury always greatly appreciated by nurses, it being understood that they wait upon each other on these occasions, and so do not add to the work of the servants. Another long-cherished plan is now to come into operation. In addition to the weekly lecture and instruction classes which have for years been given to probationers studying for their examinations, each probationer now gets one hour twice a week, from eleven to twelve noon, to write out her lecture and for study. This is arranged for in two rooms under the supervision of two home sisters. Thus the necessary study is provided for, and does not interfere with the regulation daily two hours off duty. Miss Lückes and her staff are much to be congratulated on these very important improvements, which are sure to work for the advantage of all concerned.

### Practical Points.

*"A Correspondent" writes: It has been proposed to establish a "District Nurses' Home" in W., where there is a population of between seventy and eighty thousand, largely composed of the working classes. Can you tell me how much it would cost to maintain a home for four or six nurses, and if you consider that number sufficient for the population? What would be the cost of establishing such a home?*

In order to estimate the cost of establishing a district nurses' home the local circumstances have to be borne in mind. House rent and taxes must vary with each locality, and if a suitable house be available it might be best to purchase it. The items of expenditure include the following: A capital sum for the purchase of the house and furniture and fittings, say £1,200 for the house and £200 for the furnishing, and another £100 for appliances, dressings, &c., or a rental of, say, £80 per annum, with rates, taxes, and repairs. Then the cost of maintenance; the cost of a staff of six nurses and upkeep of house would be about £480, with salaries; salary of lady superintendent £60, making with her maintenance £100; and, say, £40 for maintenance and wages of servant, and £50 for sundries; probably £700 altogether per annum when the institution gets into working order. For the number of nurses for the population, ten nurses would not be too many for seventy to eighty thousand people, though six would be a good number to begin with.

### Everybody's Opinion.

[Correspondence on all subjects is invited, but we cannot in any way be responsible for the opinions expressed by our correspondents. No communication can be entertained if the name and address of the correspondent is not given, or unless one side of the paper only is written on.]

### THE JOHANNESBURG NURSES.

Mr. H. S. Binney writes: I notice Nurse "C. E. J.'s" letter in your issue of to-day (February 27th). I have a letter from my daughter from the Johannesburg Hospital to hand by this morning's South African mail. She entirely corroborates what Nurse "C. E. J." says, saying she is not in sympathy with the nurses who left and were summoned by the Board. It is quite true they were over-worked on arrival, but she says the Board have done their best to make them comfortable, and there is no ground for complaint as to the food. She further says the Board have been very considerate to any of the nurses who wished to leave, and states that one of her friends left recently on the payment of £40 to the Board, and is now nursing private patients on her own account in Johannesburg. She says she does not think the Board would treat any of them unkindly if they were wishing to leave (and at present she has no idea of leaving), and the Board pay the return first class passage back at the end of the agreement for three years (one year of which has gone). As I write to you on the matter, I think it nothing but right that I should give you the information I have from my daughter by this mail delivered this morning.

### PRIVATE NURSING INSTITUTIONS.

A Superintendent writes: The subject of nursing and nurses is before the public very prominently just now; the December numbers of the *National Review* and *Nineteenth Century* contain articles treating this subject from slightly different points of view, and I wish to approach it from another standpoint—that of superintendent of a large private nursing staff, with some years of experience of nurses and a keen interest in all that affects them. That the profession is crowded with most unsuitable women, and that the public suffer for it, is too sadly obvious to need comment; it only remains to find the remedy. To a great extent, doubtless, it lies in the hands of matrons of training schools in their choice of probationers, but not entirely. Granted that a woman is all that she ought to be when she enters on her period of training—earnest, devoted, religious—is it not the case that in many hospitals (I do not say in all) helps to keep up the spiritual tone are wanting? Close contact with the mysteries of life and death, of sin and suffering, are a test of character, and no woman leaves the hospital quite as she entered it; she is either a much better or a much worse woman; she needs exceptional help to meet the exceptional needs during this time. I speak from personal experience as well as from what many of my own nurses have told me of the decline of spiritual life while in hospital from want of the outward help of religion. Nursing must be a religious work to be of any value at all, and the attempt to secularise will, if steps be not taken to avoid so great a calamity, end in its downfall as a power for good in the world. It is, for obvious reasons, impossible that the entire field of nursing in this country can be occupied by religious communities, but the spirit to be found in them must animate those who undertake the work, and I trust I may not be considered narrow by those differing from me when I state my conviction that a modified community life, with careful observance of the rule of the Church as regards fast and festival, and arrangements for constant access to and use of her sacraments, is not only possible to hospital and institution, but greatly minimises the likelihood that unsuitable women will seek to enter them. Another cause, I think, of the falling off in the tone and standard of the profession is that the spirit of the age, the spirit of independence and revolt against all rule and authority, has entered there. I have no wish to disparage independent effort, but I am sure that the co-operative system, while possessing many advantages, has its worse side, and that if an institution be what it ought to be and can be, it is the best system after all for securing a higher tone, and the right kind of woman will find it the most satisfactory course to join such an one. I am well aware how vehemently private nursing institutions are attacked just now, in what low estimation they are held, and how nurses are warned against them; no doubt this is all deserved in some few instances, perhaps in more than a few, but I think better of human nature and my profession than to suppose my own views and methods to be in any way uncommon or remarkable, and I believe there are a large number of institutions where the greatest care is taken in selecting nurses, not only as regards a thorough and satisfactory training, but as regards moral character and fitness for the work, where much care and thought are bestowed on the suitability of each nurse to her particular case and where the superintendent feels her responsibilities and is the friend of her nurses; to the best of her ability guarding them from unfitting work, caring for their health, and encouraging them to go out into the world as helpful, obedient workers. I would here like to touch upon a point which militates strongly against the success of institutions worked on these lines, I mean the mischief wrought by the public by depreciating the institution to the nurses, or, rather, the whole system of institution, and sowing the seeds of discontent and distrust where no cause for either exist, speaking sweepingly (and generally ignorantly) of the faults of a few as though they were found in all, and too often rendering the nurses unsettled and unhappy. If the terrible experiences of which we hear so much are common, why, may I ask, do not the public make some effort to find out where they can be supplied with steady, reliable women? One chooses one's medical adviser with care, one considers carefully the fitness of any person admitted to a position in one's household, but too often a nurse, in whose hands such serious issues lie, is chosen in a very haphazard fashion. If need be, even in those institutions which are private property, a committee of reference would be feasible, which might make the choice easier. Another matter to which attention has very properly been called, is the fee charged for and by private nurses, and the difficulties felt by those of limited means in affording to employ them, however great the need. To meet this difficulty a lower class of nurse both in training and in social position, and charging a lower fee, has been suggested. I do not agree with this; a little knowledge is a peculiarly dangerous thing in nursing. Let us have skill, education, and refinement also if possible; these last possessed by a woman of the proper spirit will make her more helpful, more obedient, more ready to undertake menial duties, not less so; this has invariably been my experience, and to remedy the difficulty in question why cannot the fees charged by institutions be on a sliding scale according to the patient's means and position? It has always been my rule to make them so, the highest being £2 2s. per week, the lowest nothing at all if the real necessity was proved, and I think there must be many other institutions where this is done, or could be done, that is, if they are really worked as useful works for the public good and not merely to make money by an individual. Needless to say, one may be "done." I have, in fact, often had that experience when the rich and mean represented their circumstances as necessitous; and great care is necessary in reducing the fee; still it can be done with a large measure of success. The want of cordial co-operation with the heads of nursing institutions displayed by the public, and I notice even by medical men, is very regrettable, and a serious hindrance to satisfactory work. The superintendent does not see her nurses at work, and is consequently very dependent on the reports she receives of them from patients and doctors. These are, as a matter of fact, too often valueless, because, from some most mistaken idea of kindness and unwillingness to injure the nurse, the truth is not stated, and the faults of the nurse concealed from the only person who could remedy them, or, remedy failing, could remove her from the work. The injury to the public is great, the superintendent in perfect good faith sending out a nurse with serious faults, of which she has no knowledge, and the consequence falling on some other patient. I do not exaggerate this; as I write I have in my mind an instance in which the nurse brought with her a very excellent testimonial, while the writer warned her friends against her immediately afterwards. It is true that it is greatly on the worth of nurses and superintendents that the value of an institution depends, but until the public see that they too have a duty in the matter the best possible standard will never be reached. The question of uniform comes forward naturally when one writes of nursing, and of the many fraudulent imitations which we see daily; and many seek to give it up. No; let the conduct of those who wear it with a right to do so be such as to gain respect until such time as it will be protected by law, which I hope is not an impossible hope in the near future. I end these remarks with the expression of my earnest conviction that in spite of all the folly, and worse than folly, of which we hear so much, there is still a vast army of earnest, pure-minded workers in the nursing profession who are not above obedience, and who know no greater ambition than to serve faithfully and tenderly the sick—"God's prisoners laid in bonds by His own hands."

### Appointments.

MATRONS.

Yeatman Hospital, Sherborne.—Miss A. L. Clark has been appointed Matron at the Yeatman Hospital, Sherborne. Miss Clark was trained at the London Hospital, and afterwards held the posts of night sister and ward sister at the Taunton Hospital. Thence she went as sister to the Derby Hospital, and for the last three and a half years has held the post of sister at the Royal Albert Hospital, Devonport, where she has been very popular, and will be much missed. Miss Clark will carry with her to her new work many hearty good wishes. Her testimonials are excellent.

Boston Hospital, Lincolnshire.—Miss L. P. Lessey has been appointed Matron of this hospital. Miss Lessey received two years' training at the London Hospital, and has since, for nearly three years, worked as charge nurse at the Boston Hospital. On the recent resignation of Miss Disney, the matron, Miss Lessey was unanimously appointed to fill the vacant post.

Malvern Isolation Hospital.—Miss Edith Holtom has been appointed Matron of this hospital. Miss Holtom trained at the City Hospital, Lodge Road, Birmingham. She has since held the post of sister in charge at the City Hospital, Little Bromwich, Birmingham.

Eastern Fever Hospital, Homerton. — Miss Mary Givens has been appointed Matron at this Metropolitan Asylums Board Hospital. Miss Givens was trained at the Brounlow Hill Infirmary, Liverpool, going thence to the City Hospital, Liverpool, as head nurse. She was subsequently promoted to the position of assistant matron at the latter institution, and then appointed matron.

Park Fever Hospital.—Miss Mary E. Jones has been appointed Matron at this new M.A.B. hospital. Miss Jones received her training at the Royal Southern Hospital, Liverpool, afterwards working at the Cardiff Infirmary and at the Monsall Fever Hospital. Latterly she has held the appointment of matron at the Eastern Fever Hospital, Homerton.

### Minor Appointments.

Newport Union Infirmary.—Miss Agnes Gameson has been appointed to the post of Night Nurse at Newport Union Infirmary. Miss Gameson was trained at the Cardiff Infirmary.

Bolton Infirmary and Dispensary.—Miss Adeline Studdard Aynsworth has been appointed Night Superintendent at this institution. Miss Aynsworth holds a three years' first-class certificate from Bolton Infirmary, and has had besides a year's gynæcological work in a private hospital at Manchester and experience in private nursing on her own account.

### Where to Go.

Trained Nurses' Club, 12, Buckingham Street, Strand, W.C.—L.O.S. preparation classes are announced twice a week at this club, on Mondays and Thursdays at four o'clock. Members can attend one lecture on payment of 2s. The subject of the week's lectures can be obtained from the secretary.

The Victoria Commemoration Club.—On Tuesday next, March 9th, at five p.m., a practical demonstration on "Small Dainty Dishes for the Sick Room" will be given at the club. The lesson will last about two hours, and will be of especial value to those in any way responsible for catering for the sick, including the making of wine jelly, sole au gratin, invalid cakes, wine whey, egg jelly, diabetic cream. Tickets for the lecture may be obtained from the secretary, price 1s. each to members and 1s. 6d. non-members. If to be sent by post a stamped addressed envelope must accompany the application. The secretary advises an early application for tickets as the space is limited.

### Notes and Queries.

**The contents of the Editor's Letter-box have now reached such unwieldy proportions that it has become necessary to establish a hard and fast rule regarding Answers to Correspondents. In future, all questions requiring replies will continue to be answered in this column without any fee. If an answer is required by letter, a fee of half-a-crown must be enclosed with the note containing the enquiry. We are always pleased to help our numerous correspondents to the fullest extent, and we can trust them to sympathise in the overwhelming amount of writing which makes the new rules a necessity. Every communication must be accompanied by the writer's name and address, otherwise it will receive no attention.**

*Monthly Nursing.*

(153) Please tell me how I can set about becoming a nurse? I want to go in for monthly as well as other training. I am 23.—*I. C.*

Read "How to Become a Nurse" (Scientific Press, 28 and 29, Southampton Street, Strand). Apply at some of the hospitals and infirmaries of which you will there find a list.

*Nurses as Stewardesses.*

(154) Please tell me what shipping company will take nurses as stewardesses? I am anxious to get such a post. I am told only those who have been to sea are eligible.—*A. E. W.*

Write to the office of the P. and O. Steamship Company, Cockspur Street, London, W. Naturally it is very necessary that anyone undertaking the work of a stewardess should be a good sailor.

*Training in the Provinces.*

(155) Can you tell me of any hospitals in the South of England where I might be admitted as a probationer? I have done temporary work in an eye hospital, and am told some hospitals object to take probationers who have had any other training.

Try at the Winchester County Hospital, Southampton Infirmary, Salisbury Infirmary, or the Kent and Canterbury Hospital. See reply to I. C. above.

*District Nurses and Bicycles.*

(156) Nurse Wilson, 3, South View, Spring Road, Wrexham, would be glad to know if any district nurse who uses a bicycle could give her an idea, or send her a pattern, of a suitable cloak?

Read what is said on "Cycles for District Nurses" in this month's "Nursing Notes," and an article on the same subject in the *Queen* for January 22nd. Perhaps some reader who has practical experience will not only reply to Nurse Wilson's request, but will send us a communication on the matter which may help other district nurses.

*Massage.*

(157) Please tell me of a cheaper book on massage than Mrs. Creighton Hale's?

Hyde's "Nurses' Guide to Massage" is published at 1s. 6d., and Ostrom's "Massage and the Swedish Movements" at 3s. 6d. Both books can be procured through the Scientific Press, 28 & 29, Southampton Street, Strand. Perhaps some of our readers may have a copy of a text-book on the subject to dispose of second-hand.

*Sanitary Inspectorships.*

(158) Please tell me if there is any opening for lady sanitary inspectors, and if so where I could obtain information concerning the examinations to be passed? I am a trained nurse, which I believe is an advantage.

Write to Miss Lamport, 52, St. John's Wood Road, N.W., on this subject.

*Nursing.*

(159) Can you tell me of a book dealing with the requirements of medical and surgical cases in hospital wards, in the way of dressings, instruments, &c?—*F. C.*

Miss Hampton's "Nursing" is an excellent text-book. The newest book on nursing is the recent edition of Miss Clara Weeks-Shaw's book, prepared for English nurses. Dr. Miles' "Surgical Ward Work and Nursing" gives full details about instruments and surgical nursing. You can get these books through the Scientific Press, 28 and 29, Southampton Street, Strand.

